# Genetic Inheritance of Female and Male Morphotypes in Giant Freshwater Prawn *Macrobrachium rosenbergii*

**DOI:** 10.1371/journal.pone.0090142

**Published:** 2014-02-26

**Authors:** Hung Dinh, Nguyen Hong Nguyen

**Affiliations:** 1 Research Institute for Aquaculture No.2, Ho Chi Minh City, Vietnam; 2 Faculty of Science, Health, Education and Engineering, University of the Sunshine Coast, Maroochydore, Queensland, Australia; The Ohio State University, United States of America

## Abstract

Giant freshwater prawn (GFP) *Macrobrachium rosenbergii* is unique with males categorized in five different morphotypes (small claw, orange claw, blue claw, old blue claw and no claw males) and females in three reproductive statuses (mature ovary, berried and spawned females). In the present study we examined genetic inheritance of female and male morphotypes, their body weights and genetic associations between morphotypes and body traits. Restricted maximum likelihood fitting a multi-trait animal model was performed on a total of 21,459 body records collected over five generations in a GFP population selected for high growth rate. The estimates of variance components showed that there were substantial differences in additive genetic variance in body weight between male morphotypes. The low and significantly different from one genetic correlations between the expressions of body weight in male morphotypes also suggest that these traits should be treated as genetically different traits in selective breeding programs. By contrast, body weights of female types are essentially the same characters as indicated by the high genetic correlations between homologous trait expressions. In addition to body weight, male morphotypes and female reproductive statuses were treated as traits in themselves and were analysed as binary observations using animal and sire linear mixed models, and logit and probit threshold models. The estimates of heritability back-transformed from the liability scale were in good agreement with those obtained from linear mixed models, ranging from 0.02 to 0.43 for male morphotypes and 0.06 to 0.10 for female types. The genetic correlations among male morphoptypes were generally favourable. Body weight showed negative genetic associations with SM (−0.96), whereas those of body weight with other male morphotypes were positive (0.25 to 0.76). Our results showed that there is existence of heritable (additive genetic) component for male morphotypes, giving prospects for genetic selection to change population structure of GFP.

## Introduction

Giant freshwater prawn (GFP) *Macrobrachium rosenbergii* is a very unique aquatic species with males categorized in five different morphotypes (small claw, orange claw, blue claw, old blue claw and no claw males) [1]–[3]. In a sexually mature population, blue claw (BC) males are generally the largest individuals having very long claws that are deep blue in colour. BC males are dominant, territorial but they grow relatively slowly. Orange claw (OC) males are also large and have long claws (but shorter than BC males) that are usually orange in colour. OC males are not territorial, have poorer mating success but are in a fast growth phase. Small claw males (SM) are small and have short claws that are generally not pigmented and are translucent. SM males are subordinate, non-territorial and only mate with females using an opportunistic reproductive behaviour. Males progress from SM to OC and BC and have a strict structure hierarchy with territorial BC males socially dominant over OC males that in turn, are dominant over SM males. Presence of BC males inhibits growth of SM males and delays metamorphosis of OC males into the BC phenotype [4], [5]. OC males can continue to grow until they are larger in size than the largest BC male in their neighbourhood before transforming into BC form [5]. In addition to the three basic male morphotypes, males that had lost their chelipeds (No claw − NC) and individuals that appeared senescent (Old blue claw − OBC) are also reported [2], [3]. Different male morphotypes have a significant effect on mean body weight and consequently they will tend to skew the distribution of body weight. In GFP, body weight and male morphotype should be thus considered and treated differently from that practised in other aquaculture species.

A number of methods have been proposed to alleviate the impacts of social population structure in commercial GFP production, for instance, by ablation of immobile leg (produs) in males [4] or improvement of culture management practises such as size grading of juveniles prior to stocking [5], supply of adequate quantity and high quality feeds [6], reduced stocking density [7] or shortened larval culture period [8]. Although positive outcomes are reported, suppression of growth via social dominance still poses huge challenge under various practical production systems of GFP farming. To maximize yield and profitability for the sector in the long term, it would be ideal to change male proportion for desired morphotypes through genetic means.

Conventional selective breeding offers several favourable attributes, especially when genetic gain is achieved, it is cumulative, permanent and sustainable and passed on from generation to generation. A number of recent studies have showed that there is low to moderate genetic variation in body traits in GFP [9], [10] and selection to improve performance traits in this species is effective [11]. Despite these success outcomes, there has not been any report on quantitative genetic basis for male morphotypes and their genetic association with body traits in this species (GFP). For instance, we still do not know whether a desired morphotype such as orange claw males can be altered by selection and if selection for the desired morphotype may cause possible correlated changes in other morphotype in freshwater prawn population. There is also no scientific information regarding the effect of male morphotype and environment interaction in *M. rosenbergii*. For example, if the expression of body traits in male morphotypes were determined to a large extent by different genes, their expressions should be treated as genetically different traits. It is therefore necessary to understand the genetic basis of male morphotypes in GFP because this source of information would provide parameters to enable the design of alternative selection strategies to improve overall performance of the population and at the same time to minimize adverse impacts on other traits.

Therefore in the present study we treated male morphotypes as traits in themselves and body weights of five different male morphotypes as genetically different traits. We estimated heritabilities for these traits as well as genetic correlations between the expressions of body weight between male morphotypes. The same approach was applied to estimate genetic parameters for female reproductive statuses.

## Materials and Methods

### Animals and data records

Data were collected from five generations of selection for high growth in a population of Giant freshwater prawn *Macrobrachium rosenbergii* at Southern Freshwater Breeding Station of the Institute for Aquaculture Research No.2 in Vietnam. A full detailed account of the population, family production, selection procedures and management of the animals are given in Hung et al. [11]. In brief, the base population (G0) was established in 2008 from a full 3×3 diallele cross involving two wild prawn stocks collected from geographically isolated rivers in Vietnam and another one imported from Malaysia through the WorldFish Centre. Two lines were formed by within- family sampling from four to 10 females and males. They came from 88 families produced from the full diallele cross in 2008. One line was selected for high breeding values for body weight (called Selection line), and another selected for breeding values of body weight as close to the population mean as possible (Control). A combined between and within family selection was practiced in the selection line. The average proportion of selected animals was about 5.1% in females and 3.3% in males. Note, however, that selection was on breeding values but not by truncation (due to the inability of some selected breeders to reproduce or due to mortality we had to resort to selecting lower ranking prawns; also, the number of selected individuals contributed by each family was restricted to avoid later inbreeding). In generations G0 (year 2008), G1 (year 2009), G2 (year 2010), G3 (year 2011) and G4 (2012) they were the progeny of 99 to 144 dams and 60 to 76 sires for the selection line. Matings were made among genetically unrelated broodstock based on their EBVs and their relationship to other animals in the pedigree to produce full-sib and (paternal) half-sib families. To produce the Control line, as many as 17 to 42 full-sib control families were produced each generation to estimate selection responses. In all generations, both the Selection and Control lines were produced and selected in parallel. The Control line was mated at the same time as the Selection line and the family production process applied identical protocols. A total of 412 sires and 545 dams successfully produced progenies used for the analyses in this study (Table 1).

**Table 1:** Pedigree structure and number of offspring recorded over generations of selection.

Generation	Sires	Dams	Offspring
G0 (2008)	81	81	1870
G1 (2009)	93	176	3771
G2 (2010)	80	116	5902
G3 (2011)	107	186	6844
G4 (2012)	101	127	3072
**Total**	**462**	**686**	**21,459**

### Family production, larval rearing, tagging and communal grow-out

Family (full- and half-sibs) production was normally completed within 30 days. Larvae from each family were reared separately in 70 l circular plastic containers (G0 to G2) and in 1 m^3^ fiberglass tanks (G3–G4). We employed an open clear water larval culture system [12] with addition of probiotics. Larvae were fed only with newly hatched brine shrimp nauplii (3 times per day) for the first ten days followed later by a combination of brine shrimp nauplii and egg custard (chicken egg, high calcium milk powder, shrimp, squid flesh and fish oil) [13]. Post-larvae (PL) were normally observed after 20 to 30 days in larval rearing tanks and metamorphosed into the PL stage after 25 to 40 days. Post-larvae from each family were reared separately in 1 m^3^ fiberglass tanks for two weeks at a stocking density of 1,000 PLs per m^3^. They were fed with a commercial prawn pellet (available for *P. monodon*) at starting feed size. After 2 weeks, families were transferred separately into fine mesh hapas of 4 m^2^ submerged into an earthen pond at a stocking density of 150 individuals per m^2^. Hapas were supplied with air from 9 pm to 6 am and PLs were fed with a 40% crude protein commercial prawn pellet (manufactured by Uni-President Co. in Vietnam). PLs were kept in hapas for six to eight weeks until they reached a suitable size for tagging (around 2 g). All juveniles in each family were tagged as a batch using visible implant elastomer (VIE) tags as described by Hung et al. [14]. Two tags of 5 to 6 different colors were applied to individual prawns to maintain pedigree records. After tagging, juveniles from each family were kept in a 1 m^3^ fiberglass tank supplied with constant aeration and fed with pellets for 3 days to acclimate. After tags were verified, 120 juveniles chosen randomly from each family were released into two common earthen ponds of 3,500 m^2^ for grow-out. Grow-out stocking density was set at 2 individuals per m^2^. No aeration was required and environmental factors were checked frequently. Water was exchanged at least twice a month via gravity flow or by pumping when required. Juveniles in grow-out ponds were fed with a commercial prawn pellet containing 35% crude protein at stocking that was reduced to 30% for the last 4 grow-out weeks. In addition to the high quality diet and low stocking density, substrate provided to reduce cannibalism and social interaction effects included 20 cm pieces of 100 mm PVC pipe and bamboo branches. Grow-out time was set for 18 weeks (market age) so that the total culture time was 26 to 28 weeks from PL to harvest as is the case in commercial production. At harvest, all individuals were harvested using a cast-net and measurements were taken as follows.

### Trait measurements

At harvest six body traits (body weight, body length, cephalothorax length, abdominal length, cephalothorax width and abdominal width) and three carcass weight traits (abdominal weight, skeleton-off weight, telson-off weight) were measured on each individual. Animal identification (i.e. tag code), sex, culture pond, measurement dates were also recorded at harvest.

In addition to body and carcass weight trait records, five adult morphotypes for males and three individual reproductive status classes for females were recorded. For males, three basic morphotypes that have been documented by Kuris et al. [1] include: blue claw males (BC), orange claw (OC) and small (SM) males. In addition to the three basic male morphotypes, males that had lost their chelipeds (No claw − NC) [15] and individuals that appeared senescent (Old blue claw − OBC) [3] were also scored. Hulata et al. [15] reported that NC males were mainly BC males that had underwent cheliped autotomy. We also observed NC males that resulted from fighting with other males or that occurred due to netting or handling during harvest or measurement. In nature, NC males can regenerate new orange claw(s) and will continue to grow. OBC males are believed to evolve from BC males, they have a senescent appearance and their bodies are covered with algae.

Female GFP can be classified according to their reproductive status. For mature females, Ra'anan et al. [16] classified them in two groups: 1) Berried female (BF) that are females with an egg mass suspended under the abdomen, ii) Spawned females (SF) that are females that had already spawned. Beside the two mature female groups described, we also found post-ovigerous females, with an enlarged brood chamber and having visible gonads which we called mature ovary females (MOF). This female GFP classification system was also applied by Thanh et al. [13] and Aflolo et al. [17].

### Statistical analysis

#### Morphotypes

Male morphotypes and female reproductive statuses were treated in the form of ‘presence’ or ‘absence’ (‘yes’ or ‘no’) and were coded as 1 or 0. Due to binomial characteristics of these traits that are not normally distributed and the assumption of homogeneity of variance and linearity are not met, they were analysed using generalized linear mixed model (GLMM) with a logit and probit link functions. In addition, male morphotypes and female reproduction statuses were also analysed using standard linear mixed models (LMM). In total, four different statistical models were used, namely linear animal model (model 1), linear sire model (model 2), logit threshold model (model 3) and probit threshold model (model 4). Goodness of fit of the statistical models was assessed on the basis of their logarithmic likelihood (LogL) or deviance values. There were only trivial differences in LogL values between models 1 and 2 or in deviance between models 3 and 4. Correlations of family breeding values between models were also calculated and all the estimates were high (close to one), indicating that both linear and threshold models can be used interchangeably. For completeness, results from the four models were presented.

The same fixed effects were fitted in the four models. They included generation (spawning seasons, 1 to 5), line (selection or control), pond (two grow-out ponds used per generation), the second order interaction between generation and line, and interaction between pond and generation. A linear covariate (number of days from stock to harvest) was fitted within sex, ponds and generation subclasses. All these effects were statistically significant (P<0.05 to 0.001). The models used here were the same as formulated by Hung et al. [11], with one exception was that male morphotypes fitted in males and female reproduction classes fitted in females were dropped from the models since they were treated as traits and analysed separately in the present study.

For linear animal model (model 1), the random term included only additive genetic effect of individual animal in the pedigree. The common full-sib effect on morphotype traits was omitted from the final model since this effect tested by logarithmic likelihood ratio (LRT) was not significant (P>0.05). The LRT test compared twice the difference in logarithmic likelihood between the full and reduced models with Chi-square distribution having one degree of freedom. Under the animal model, heritabilities for traits were calculated as 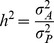, where the phenotypic variance (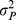) was the sum of the additive genetic variance (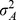) and the residual variance (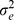), i.e. 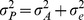.

With linear sire model (model 2), heritabilities for traits were calculated as 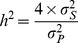, where the phenotypic variance (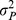) was the sum of the sire variance (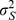) and the residual variance (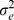).

Logit threshold model (model 3)

In addition to linear animal and sire models (1 and 2) as described above, male morphotypes and female reproductive statuses were also analysed by GLMM sire model. Under this model, the assumption was that the data followed a binomial distribution, and a logit link function (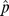 =  e^x^/(1 +e^x^)) was used where p is the probability of a male morphotype (or female reproduction class) recorded at harvest. The fixed effects fitted were the same as models 1 and 2 described above, but the random effect was sire. Threshold models analyses are normally based on sire model because the binary characteristics (0/1) of the data tend to cause statistical difficulties in animal models, e.g. Sorensen and Gianola, [18]. With GLMM sire model, heritability was calculated using the variance of the logit link function, which implies a correction of the residual variance by factor π^2^/3.
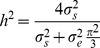
Where 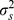 is sire variance and 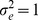


Probit threshold model (model 4)

The threshold sire model is basically the same as those described above. However, the probit link function 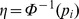 is used, with inverse link 
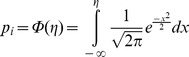
, where 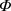 is the cumulative normal density function, and *p_i_* denotes the probability of male (female) morphotype for prawn *i*. The Bernoulli distribution for a male morphotype or female reproduction class for an individual prawn with y_i_  = 1 (presence of a given morphotype) and y_i_  = 0 (absence) is the probability (y_i_|p_i_)  =  (p_i_)y_i_(1-p_i_)^1−y^_i_.

Under probit threshold model, heritability was calculated as
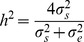
where 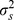 is sire variance and 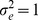.

For binomial observations, estimates of *h^2^* on the liability scales (logit and probit) can be transformed to observed (0/1) scale using the formula of Robertson and Lerner [19] as follows:
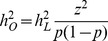
where 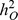 is the heritability on the observed (0/1) scale, s is the estimated heritability on the liability (logit or probit) scale, *p* is a proportion of morphotypes in the data, and *z* is the height of the ordinate of normal distribution corresponding to a truncation point applied to *p* proportion of morphotypes.

#### Body weight

In this study, body weights of male morphotypes and of females were treated as different traits and they were analysed separately. The same model as described above was used to analyse the expressions of body weight in male morphotypes and female reproductive groups. All significant fixed effects identified from general linear model analysis were fitted in the final model; they included generation (spawning seasons, 1 to 5), line (selection or control), pond (two grow-out ponds used per generation), the second order interaction between generation and line, and interaction between pond and generation. A linear covariate (number of days from stock to harvest) was fitted within sex, ponds and generation subclasses. The random effects included only the additive genetics of individual animal or in addition to the maternal and common full-sib effects (i.e. the effect of dam).

Genetic correlations between the expressions of body weights in male morphotypes were estimated through genetic relationships in the full pedigree. There were no environmental covariances between the homologous traits since they were phenotypically measured on different animals.

Genetic and phenotypic correlations between body weight and male morphotypes are estimated using a multi-trait linear mixed model. All computation was conducted in ASReml package [20].

## Results

### Descriptive statistics

**Figure 1:**
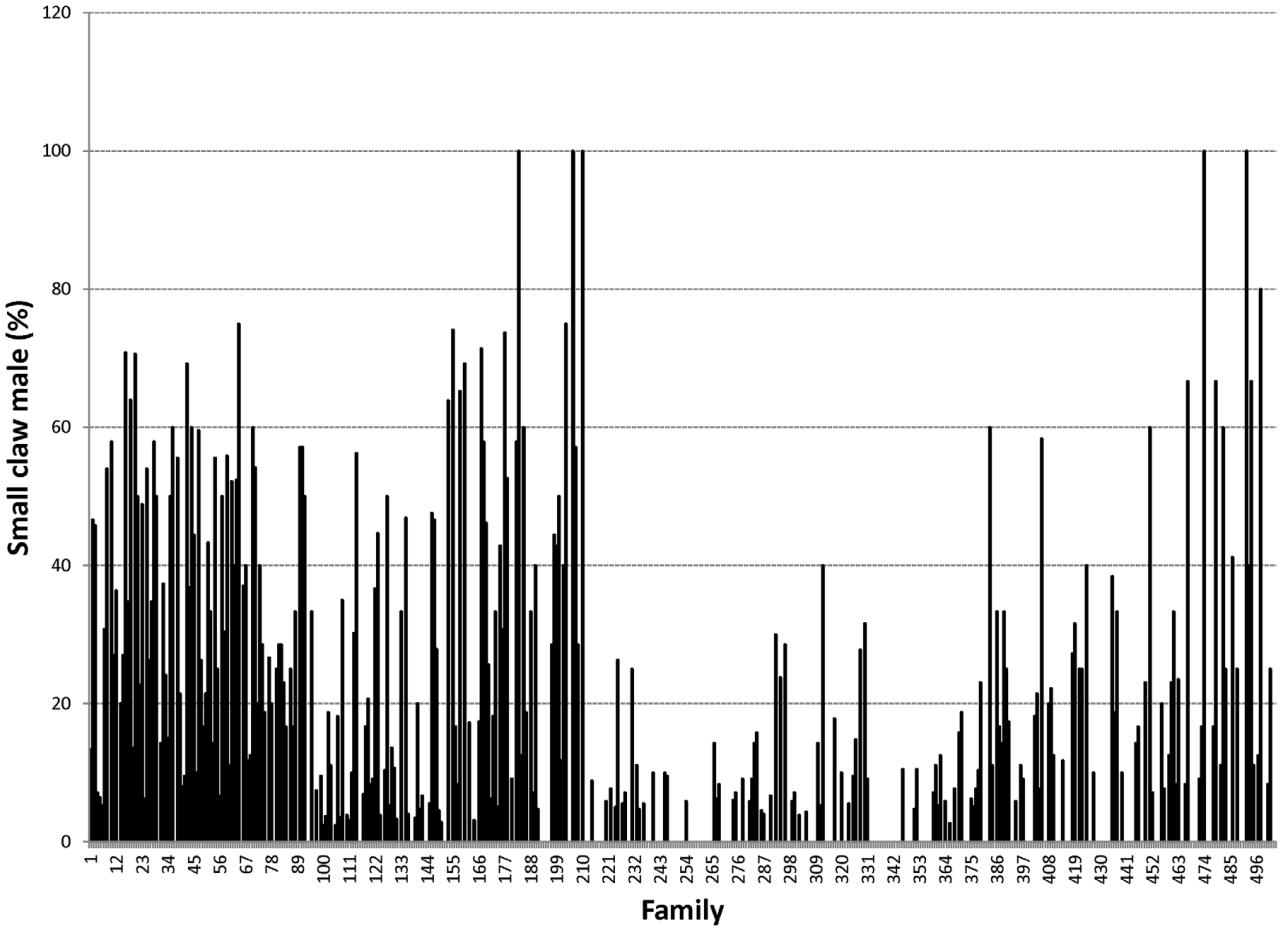
Small claw male morphotype.

**Figure 2:**
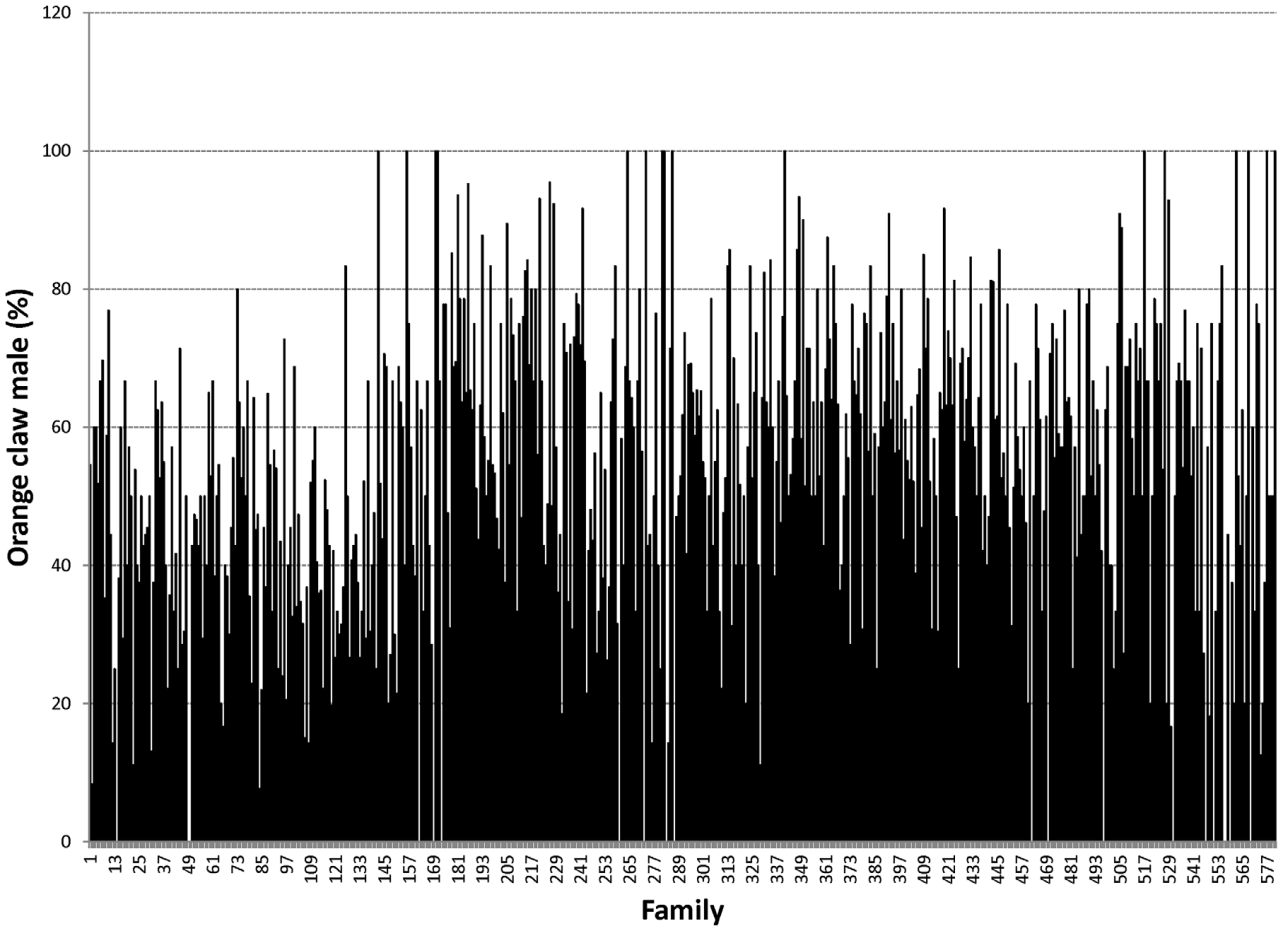
Orange claw male.

**Figure 3:**
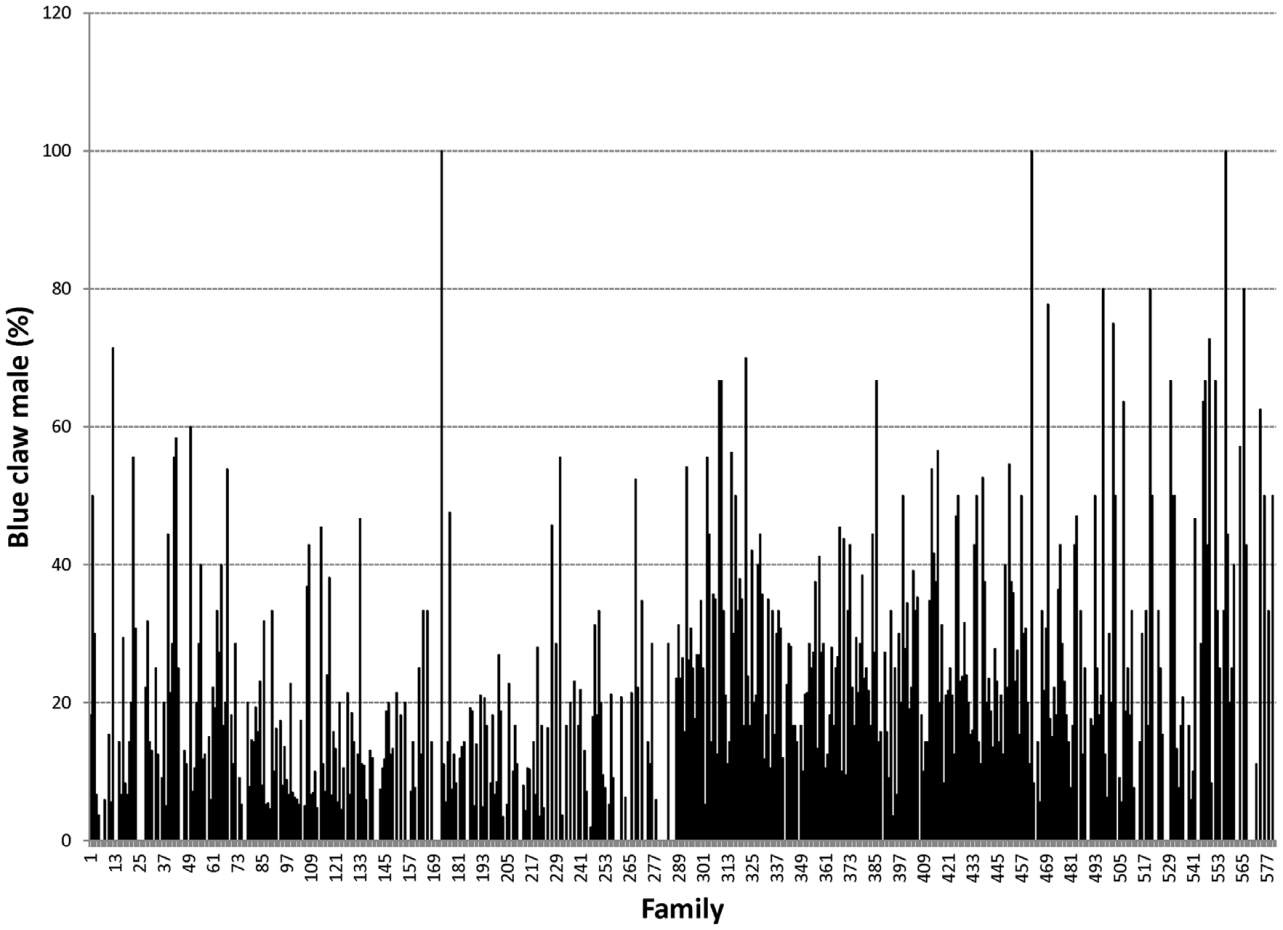
Blue claw male.

**Figure 4:**
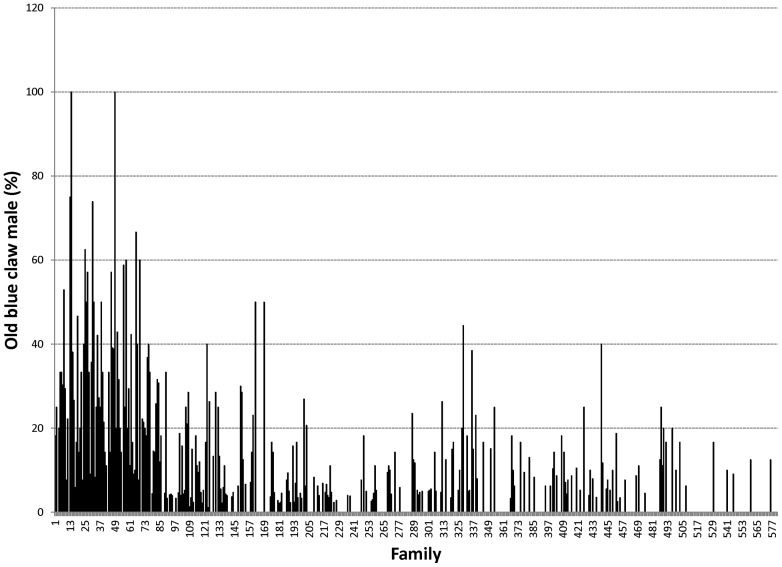
Old blue claw male.

**Figure 5:**
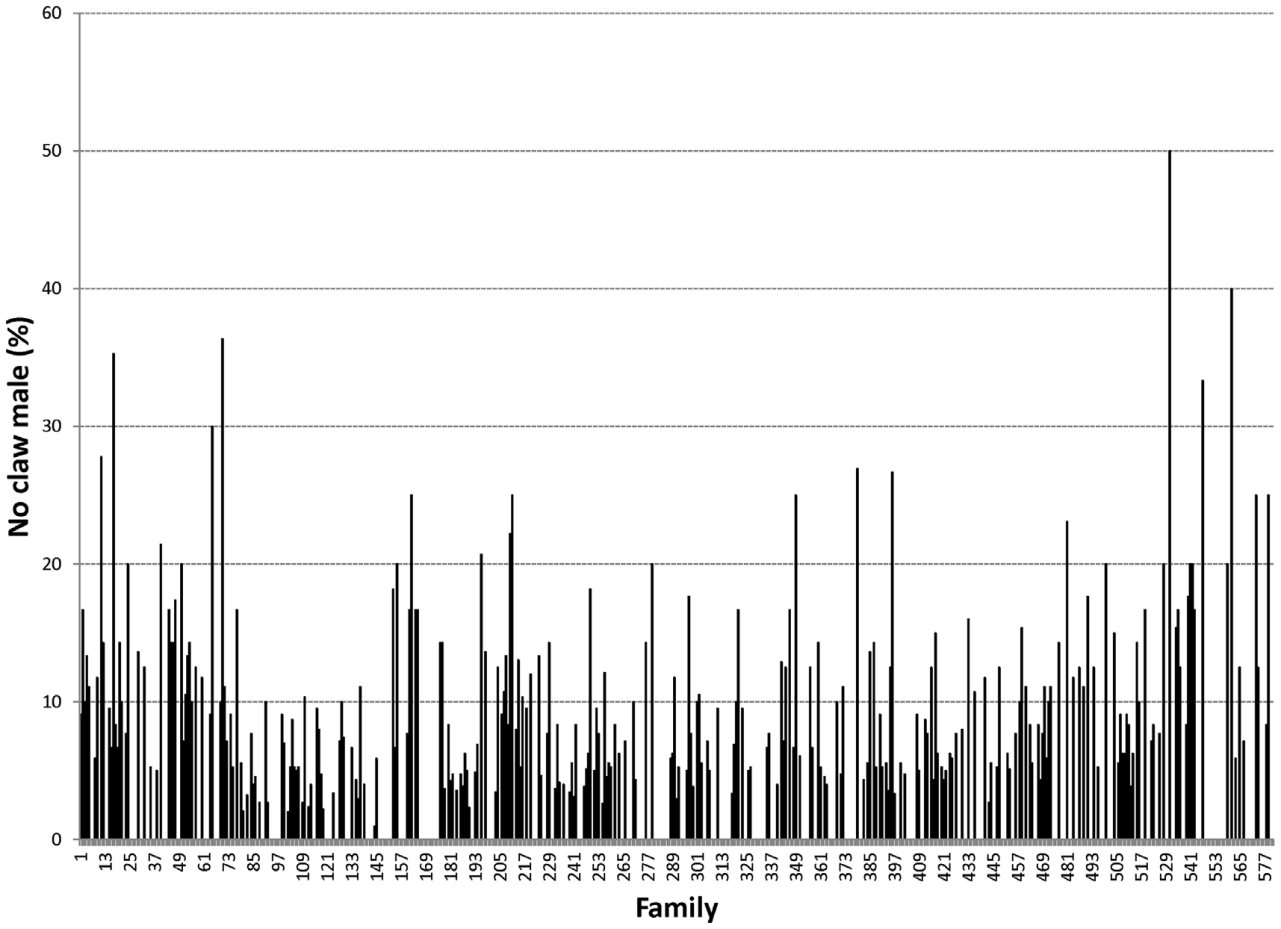
No claw male.

**Figure 6:**
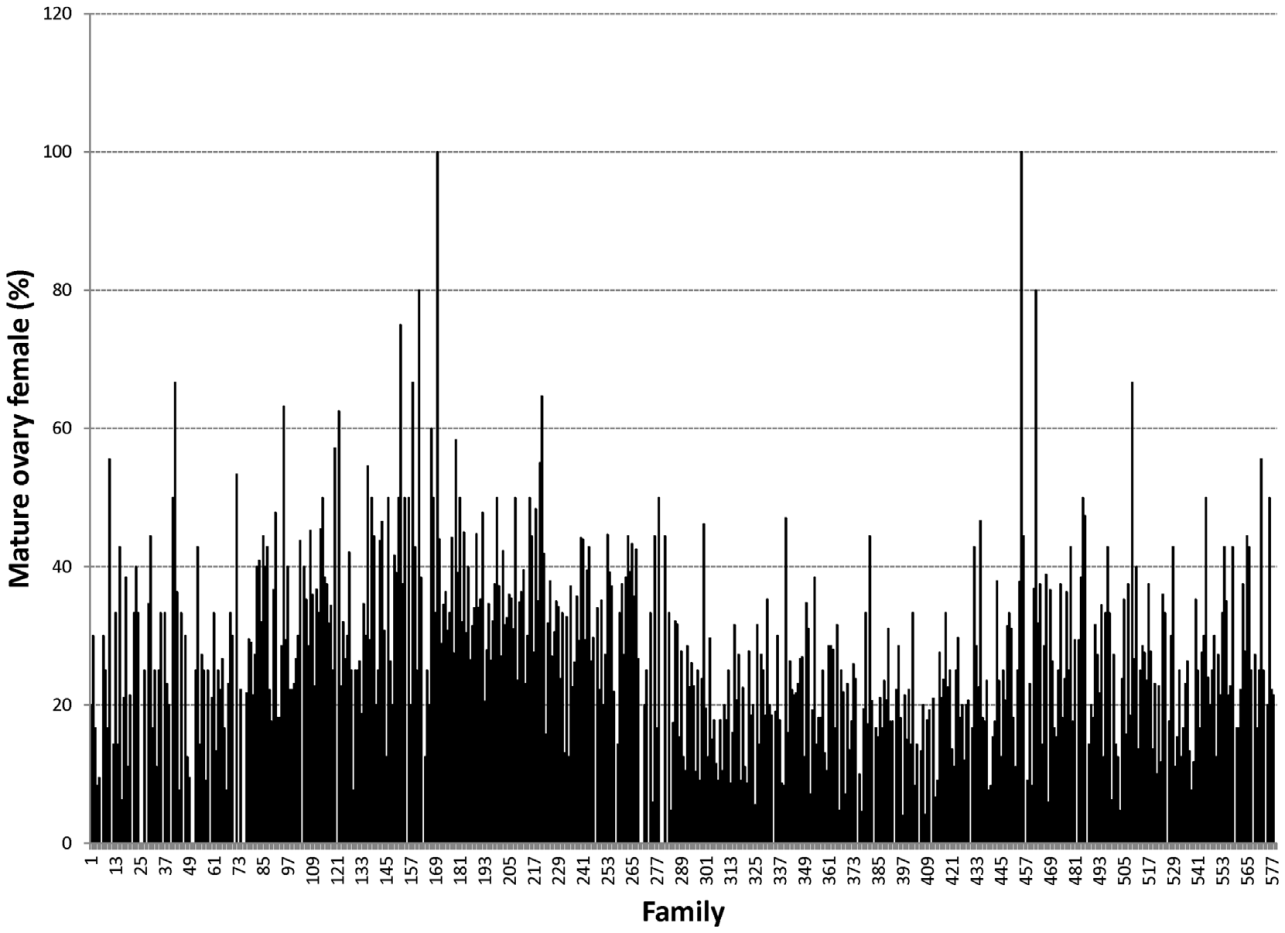
Mature ovary female.

**Figure 7:**
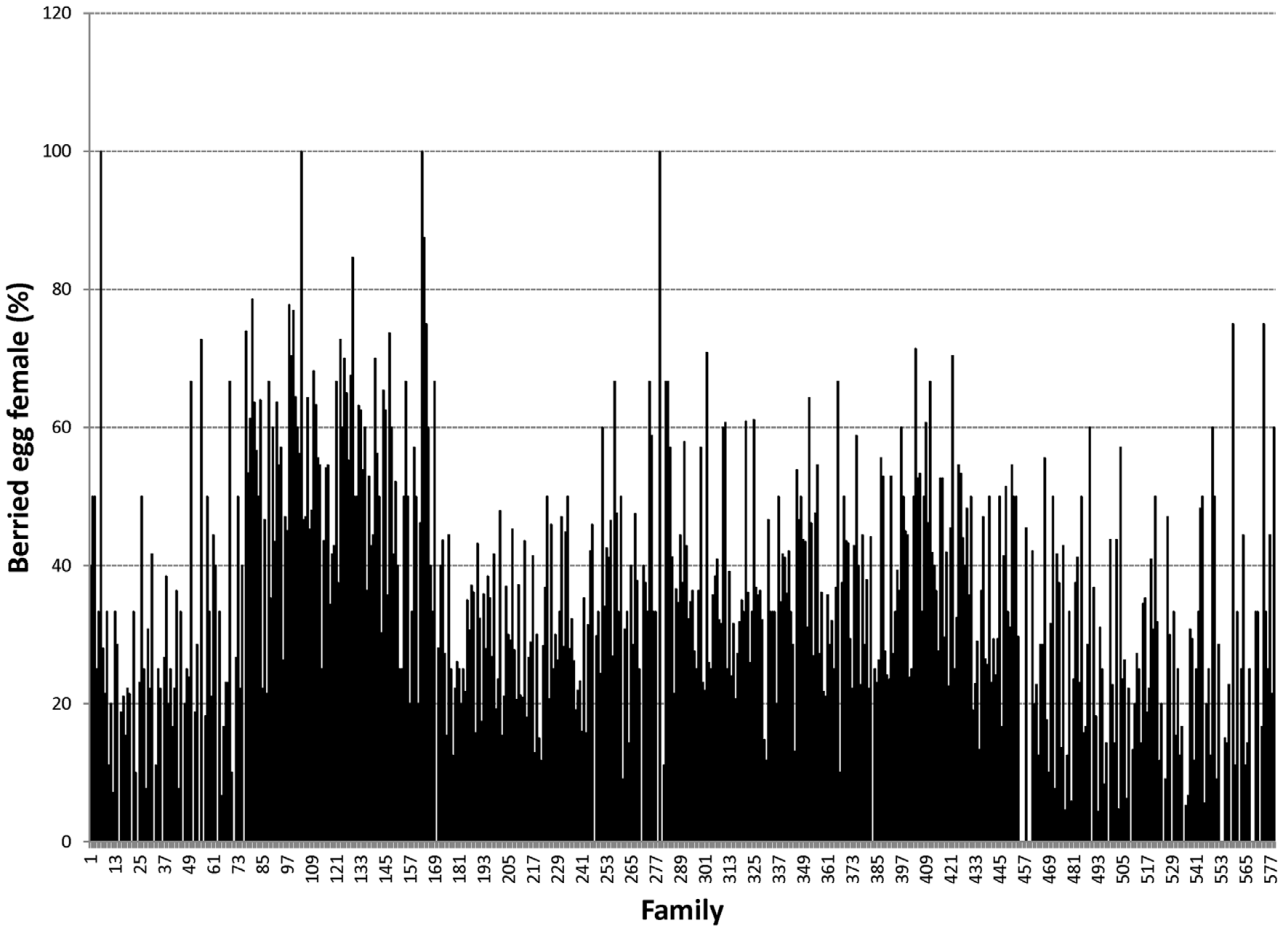
Berried egg female.

**Figure 8:**
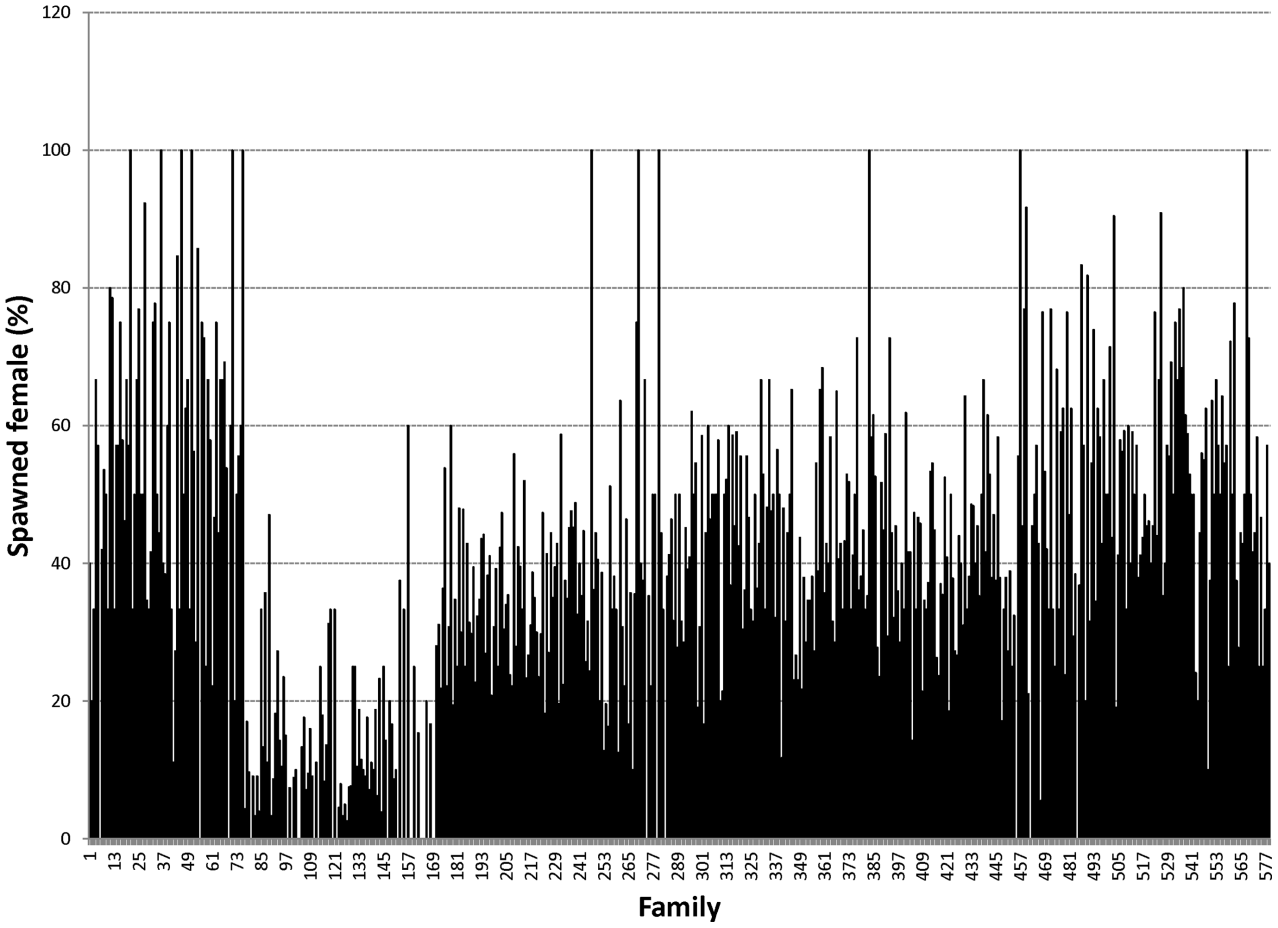
Spawned female.

Proportions of male morphotypes, females groups categorised according to their reproduction statuses and their body weights are given in Table 2. For males, orange claw morphotype (OM) accounted for the largest proportion (54%), followed by blue (18%) and small claw males (16%). Both old blue claw (OBC) and no claw (NC) males made up 12% of the total male morphotypes. There was almost no difference in proportion of females categorized according to their reproductive statuses. Variation in proportions of male morphotypes and female reproductive statuses among 576 full- and half-sib families produced between 2008 and 2012 is graphically shown in Figures 1, 2, 3, 4, 5, 6, 7, 8. Both Chi-square test and Wald F statistic showed significant differences (P<0.05 to 0.001) in composition of male morphotypes among families within the same pond and generations or across ponds and generations. For example, the proportion of SM, OC, BC, OBC and NC in one grow-out pond in the latest generation (2012) was 17.2, 52.2, 23.1, 1.2 and 6.2%, respectively. Across ponds and generations, there was also a large variation in male morphotype among families, ranging from 1.1 to 100.0% for BC or 1.2 to 98.8% for SM.

**Table 2:** Descriptive statistics for male morphotypes (%), female reproductive statuses (%) and their body weight (g).

Trait	N	Mean	SD	Minimum	Maximum
SM	9561	15.9	36.6	0	100
OC	9561	54.3	49.8	0	100
BC	9561	17.9	38.3	0	100
OBC	9561	6.9	25.5	0	100
NC	9561	4.9	21.6	0	100
MOF	11898	27.5	44.6	0	100
BF	11898	34.9	47.7	0	100
SF	11898	37.6	48.4	0	100
Weight of SM	1515	9.1	4.3	2.2	22.2
Weight of OC	5184	60.2	26.1	3.7	130.9
Weight of BC	1707	81.2	27.5	11.4	169.7
Weight of OBC	686	40.0	32.2	5.0	100.0
Weight of NC	469	55.8	24.9	6.9	110.4
Weight of MOF	3282	29.5	10.1	6.3	79.1
Weight of BF	4153	28.8	9.5	3.2	59.6
Weight of SF	4463	27.7	10.5	2.2	64.1

**SM  =  Small male, OC  =  Orange claw male, BC  =  Blue claw male, OBC  =  Old blue claw male and NC  =  No claw; MOF  =  Mature ovary female, BF  =  Berried female and SF  =  Spawned female.**

Consistent with expectations from biology of this species, blue claw (BC) males had greatest body weight at harvest, whereas small male (SM) had smallest body weight. There was about eight times difference in body weight at harvest between the BC and SM morphotypes. Body weights of three female types as categorised by their reproduction statuses were almost similar (Table 2).

### Heritability for male morphotypes and female reproductive statuses

In the present study, male morphotype and female reproductive status were also treated as traits in themselves. Heritabilities (h^2^) for male morphotypes and female reproductive status were estimated using four different statistical models (Table 3). The linear mixed model (LMM) h^2^ estimated from animal or sire as the random effect (models 1 and 2) varied largely with morphotypes, being lowest for NC (0.02) and highest for SM (0.56). The low estimate for NC was likely because of the subjective nature of recording this morphotype and its relatively low frequency in the male population. Heritabilities for OC, BC and OBC were intermediate (0.12 to 0.18).

**Table 3:** Heritability (±s.e.) for male morphotypes and female reproductive statuses estimated with four different statistical models.

Sex	Traits	Model		σ_A_^2^ (σ_S_^2^)	σ_E_^2^	h^2^ (underlying)	h^2^ (observed)
Male	SM	1	Animal	0.0733	0.0584	0.56±0.04	
		2	Sire	0.01530	0.0981	0.54±0.05	
		3	Logit	1.041	1	0.96±0.09	0.42
		4	Probit	0.315	1	0.98±0.09	0.43
	OC	1	Animal	0.0357	0.196	0.15±0.02	
		2	Sire	0.0123	0.214	0.22±0.03	
		3	Logit	0.2350	1	0.27±0.04	0.17
		4	Probit	0.0892	1	0.33±0.05	0.21
	BC	1	Animal	0.0241	0.113	0.18±0.02	
		2	Sire	0.735×10^−2^	0.122	0.20±0.03	
		3	Logit	0.348	1	0.38±0.05	0.10
		4	Probit	0.108		0.39±0.05	0.10
	OBC	1	Animal	0.0089	0.0491	0.15±0.02	
		2	Sire	0.421×10^−2^	0.0530	0.29±0.04	
		3	Logit	0.649	1	0.66±0.09	0.20
		4	Probit	0.161	1	0.55±0.08	0.17
	NC	1	Animal	0.518×10^−3^	0.0454	0.015±0.007	
		2	Sire	0.364×10^−3^	0.0456	0.034±0.016	
		3	Logit	0.121	1	0.14±0.08	0.03
		4	Probit	0.0239	1	0.09±0.06	0.02
Female	MOF	1	Animal	0.225×10^−4^	0.187	0.001±0.003	
		2	Sire	0.117×10^−7^	0.187	n.e.	
		3	Logit	0.287×10^−4^	1	n.e.	
		4	Probit	0.224×10^−3^	1	0.001±0.002	
	BF	1	Animal	0.686×10^−2^	0.205	0.03±0.01	
		2	Sire	0.390×10^−2^	0.208	0.07±0.02	
		3	Logit	0.089	1	0.10±0.02	0.06
		4	Probit	0.0326	1	0.13±0.07	0.08
	SF	1	Animal	0.0107	0.1994	0.05±0.01	
		2	Sire	0.486×10^−2^	0.204	0.09±0.02	
		3	Logit	0.112	1	0.13±0.03	0.08
		4	Probit	0.414	1	0.16±0.03	0.10

1 = LMM animal model, 2 =  LMM sire model, 3  =  GLMM sire model using logit link function, and 4 =  GLMM sire model using probit link function.

σ_A_^2^  =  Genetic variance (model 1), σ_S_^2^  =  Sire variance (models 2, 3 and 4) and σ_E_^2^  =  Environmental variance, n.e.  =  non-estimable.

Trait abbreviation in Table 1.

The generalized linear mixed model (GLMM) estimates of heritability for male morphotypes used logit and probit models. The h^2^ obtained from logit model (model 3) were slightly different from those estimated using probit model (model 4). However, note that they cannot be directly compared because the estimates of heritability from the logit model were on the logistic scale whereas the ones obtained from the probit model were on the underlying normal scale. For probit model, the highest heritability was for SM (0.96) and lowest for NC (0.14). A similar pattern was also observed for the probit models. As expected from the theory, the GLMM estimates of heritability for male morphotypes on the liability scale were remarkably higher than those from LMM (ranging from 0.13 to 0.98 vs. 0.02 to 0.56, respectively). When the estimates on logit and probit liability scales were transformed to observable scale, heritabilities were almost similar between the LMM and GLMM methods (Table 3).

### Phenotypic and genetic correlations among male morphotypes and female reproductive statuses

Phenotypic and genetic correlations among male morphotypes and female reproductive status are given in Table 4. Small male (SM) showed moderate to high and negative genetic correlations with other morphotypes. OC morphotype had negative genetic correlations with BC and OBC, but positive relationship with NC. The genetic correlations of BC with OBC and NC were high and positive.

**Table 4:** Phenotypic (above) and genetic (below the diagonal) correlations (±s.e.) among male morphotypes and female reproductive statuses.

Traits	Male					Female		
	SM	OC	BC	OBC	NC	MOF	BF	SF
SM		−0.49±0.01	−0.20±0.01	−0.49±0.01	−0.10±0.01			
OC	−0.62±0.05		−0.51±0.01	−0.29±0.01	−0.24±0.01			
BC	−0.60±0.06	−0.20±0.09		−0.11±0.01	−0.12±0.01			
OBC	−0.40±0.05	−0.14±0.09	0.26±0.10		−0.08±0.01			
NC	−0.66±0.15	0.33±0.18	0.55±0.20	−0.16±0.20				
MOF							−0.48±0.01	−0.47±0.01
BF						−0.92±0.57		−0.55±0.01
SF						−0.95±0.72	−0.93±0.13	

### Heritability for body weight of male morphotypes and of female reproductive statuses

Heritability (h^2^) and maternal and common environmental effects (c^2^) for body weights of male morphotypes and female reproductive statuses were estimated from animal and common environmental models (Table 5). The h^2^ estimates obtained from animal model (model 1) were moderate to high for male morphotypes (0.13–0.59). The estimates of heritability for body weights of male morphotypes were substantially lower (ranging 0.07 to 0.17) when the common full-sib effect was included as the second random term in the model (model 2 in Table 5). Across male morphotypes, the maternal and common environmental effects (c^2^) accounted for 7 to 31% of total phenotypic variation in body weight.

**Table 5:** Heritability (h^2^ ±s.e.) and maternal and common full-sib effects (c^2^) for body weight of male morphotypes and female reproductive statuses.

Sex	Trait	Model	WT					WT^0.5^				
			σ_A_^2^	σ_C_^2^	σ_E_^2^	h^2^	c^2^	σ_A_^2^	σ_C_^2^	σ_E_^2^	h^2^	c^2^
Male	SM	1	14.7		56.4	0.21±0.07		0.17		0.65	0.22±0.07	
		2	2.32	33.3	52.2	0.03±0.08	0.28±0.06	0.06	0.13	0.63	0.08±0.08	0.16±0.05
	OC	1	143.3		294.8	0.33±0.04		0.60		1.33	0.31±0.04	
		2	24.2	43.3	346.9	0.06±0.03	0.10±0.02	0.10	0.18	1.55	0.06±0.02	0.10±0.02
	BC	1	66.5		174.8	0.28±0.06		0.21		0.57	0.27±0.06	
		2	24.9	25.0	187.6	0.11±0.06	0.11±0.04	0.07	0.09	0.61	0.09±0.06	0.11±0.04
	OBC	1	113.7		78.5	0.59±0.12		0.30		0.43	0.42±0.11	
		2	23.0	58.6	165.8	0.12±0.13	0.31±0.07	0.05	0.21	0.47	0.08±0.12	0.29±0.07
	NC	1	44.6		302.5	0.13±0.10		0.28		1.45	0.19±0.13	
		2	22.1	30.8	294.7	0.06±0.11	0.09±0.09	0.014	0.17	1.42	0.08±0.12	0.10±0.09
Female	MOF	1	38.5		16.1	0.71±0.05		0.31		0.13	0.70±0.05	
		2	27.0	4.3	21.0	0.52±0.07	0.05±0.02	0.23	0.03	0.17	0.54±0.07	0.07±0.02
	BF	1	33.3		22.2	0.60±0.05		0.28		0.18	0.61±0.05	
		2	16.2	6.3	29.5	0.31±0.06	0.12±0.02	0.15	0.05	0.23	0.35±0.06	0.11±0.02
	SF	1	24.9		30.0	0.45±0.04		0.22		0.26	0.46±0.04	
		2	10.6	5.9	35.9	0.20±0.05	0.11±0.02	0.10	0.05	0.31	0.22±0.05	0.11±0.02

σ_A_^2^  =  Additive genetic variance, σ_C_^2^  =  Maternal and common full-sib variance and σ_E_^2^  =  Environmental variance.

The heritabilities for body weights of females were moderate to high across models (0.20 to 0.71), and generally greater than those estimated in males. In both sexes, there were substantial differences in genetic variance and consequently heritabilities between morphotypes or reproductive statuses.

### Genetic correlations between the expressions of body weight in male and female

Genetic correlations between expressions of body weight in male morphotypes and female reproductive groups are presented in Table 6. The genetic correlation estimates of homologous body weights between female reproductive statuses were high (>0.80). For males, the genetic correlations between the expressions of body weight especially between small male and other male morphotypes were low (0.32 to 0.39). There are two exceptions of moderate genetic correlations for body weight expressions between orange and blue (or old blue) clawed males (0.61 and 0.71, respectively). The results suggest that body weights of three reproductive statuses in females are essentially the same traits, whereas body weights of male morphotypes are under different genetic control and therefore should be treated as genetically different traits.

**Table 6:** Genetic correlations (±s.e.) between expressions of body weight in male and female.

Trait	Male				Female		
	SM	OC	BC	OBC	MOF	BF	SF
**SM**							
**OC**	0.27±0.52						
**BC**	0.19±0.42	0.66±0.46					
**OBC**	0.29±0.19	0.68±0.61	0.29±0.53				
**NC**	0.26±0.16	0.73±0.66	0.14±0.46	0.26±0.15			
**MOF**							
**BF**					0.71±0.49		
**SF**					0.95±0.35	0.76±0.27	

### Correlations of male morphotypes and female reproductive statuses with body traits

Shown in Table 7 are the phenotypic and genetic correlations of male morphotypes and female reproductive status with body weight. Both phenotypic and genetic correlation estimates were significantly different from zero (P<0.05). The genetic association between SM and body weight was strongly negative (−0.95). On the other hand, the remaining male morphotypes had moderate to high and positive genetic correlations with body weight (0.25 to 0.76). For females, the genetic correlations of MOF and BF with body weight were positive (0.70 to 0.84), whereas it was negative between BF and body weight (−0.86).

**Table 7:** Genetic (r_G_) and phenotypic (r_P_) correlations (±s.e.) of male morphotypes and female reproductive statuses with production traits.

Sex	Traits	Weight		Length	
		r_G_	r_P_	r_G_	r_P_
Male	SM	−0.95±0.02	−0.65±0.01	−0.98±0.01	−0.77±0.01
	OC	0.25±0.14	0.17±0.01	0.34±0.11	0.30±0.01
	BC	0.70±0.10	0.32±0.01	0.70±0.09	0.22±0.01
	OBC	0.40±0.13	0.05±0.01	0.40±0.10	−0.01±0.01
	NC	0.76±0.14	−0.01±0.01	0.72±0.16	0.07±0.01
Female	MOF	0.70±0.67	−0.04±0.02	−0.93±0.54	−0.12±0.01
	BF	0.84±0.06	0.54±0.01	−0.27±0.16	−0.10±0.02
	SF	−0.86±0.05	−0.52±0.01	−0.46±0.12	−0.11±0.01

## Discussion

Our study is the first demonstrating that there is heritable genetic component for male morphotypes in GFP (*Macrobrachium rosenbergii*). Genetic inheritance of body weight also differed among male morphotypes. The genetic correlations between the expressions of body weight in male morphotypes were all significantly different from one (022–0.71), suggesting that the interaction of morphotype and environment may be of biological importance in this species. Of particular interest, genetic basis of male morphotypes is different from female reproductive statuses. Heritabilities for three different categories of female reproduction were of similar magnitude and the genetic correlations between trait expressions in females were close to unity. The genetic correlations among male morphotypes and their associations with body traits also enable the prediction of possible changes resulted from selection for one trait or another. In both sexes, the heritability estimates back-transformed from logit and probit liability scales were in good agreement with those on original (0, 1) scales for all traits studied, indicating that both threshold and linear models can be used interchangeably to analyse prawn morphotypes. Key findings obtained from the present study are discussed in the following sections.

### Genetic basis of male morphotypes

One predominant finding we demonstrated in the present study was that there is substantial additive genetic component for male morphotypes, suggesting the prospects to change population structure of *Macrobrachium rosenbergii* through selection for desired male morphotypes. From commercial perspectives, increase in OC is desired since this morphotype (OC) has fast growth rate and is not territorial, and possessing higher abdominal carcass weight than OBC and similar abdominal weight to BC [13]. Note that both BC and OBC had greater body weight than OC. The presence of BC in mature GFP populations is generally unfavourable due to its dominance hierarchy and agonistic behaviour that suppress growth of other male groups in the population. In the present study, the heritabilities for separate male morphotypes are greater than those estimated in our earlier report [11] where male morphotypes were analysed together using animal model. In both studies we found that there were substantial genetic variation especially for SM and BC male morphotypes. The estimates of genetic correlations also showed that selection for one male morphotype may lead to changes in others. For instance, selection for increased orange claw male is expected to be associated with a reduction in small and blue claw male morphotypes. The genetic correlations among male morphotypes are as expected and are favourable from genetic improvement's perspectives.

### Morphotype and environment (M×E) interaction

The low genetic correlations for the expressions of body weight between male morphotypes in our present study also suggest that the effect of morphotype and environment (M×E) interaction could be significant [21]. The M×E interaction in this study resulted from both scaling and re-ranking effects. The additive genetic variance component for the expressions of body weight differed between male morphotypes. The genetic correlations between homologous traits were generally low. These results suggest that male morphotypes and environment interaction is of biological importance in this species. The significant M×E interaction would affect the re-ranking and selection decision of breeder candidates. Body weights of male morphotypes therefore should be treated as different traits in genetic evaluation programs. Our calculations using selection index theory pointed out that the presence of M×E interaction due to scaling effect (i.e. the difference in heritability between male morphotypes) resulted in little changes in the underlying components of genetic gains (results not shown). By contrast, the M×E interaction that results in re-ranking effect often had large impact on accuracy of selection. Changes in genetic gains for traits studied were proportional to the magnitude of the genetic correlation estimates. A decrease in accuracy of selection was the main source of loss in genetic gain. For instance, selection for body weight of blue claw male only captured 66% of genetic expression in orange claw male morphotype. In practical genetic improvement programs for *Macrobrachium rosenbergii*, the effect of male morphotype by environment interaction should be accounted for to increase accuracy of selection and consequently genetic gain.

### Genetic associations between body weight and male morphotypes

In current breeding programs for GFP, body weight has been the sole selection criterion. Our estimates of the genetic correlation between body weight and male morphotypes also showed that selection for increased body weight may be associated with a decrease in SM but increase in other male morphotypes. A reduction in proportion of SM and enhancement of OC are favourable, whereas the increase in BC may be associated with competitive social effects. The set of genetic parameters obtained in the present study for male morphotypes and body weight were used to model alternative selection strategies to increase body weight and decrease BC male morphotype. We used desired gain selection index approach to examine three simplified scenarios: 1) The breeding objective included only body weight although both the objective trait and BC morphotype were measured, 2) The breeding objective for GFP was to improve body weight while achieving no changes in BC morphotype, and 3) the objective was to increase both body weight at a predetermined change of −10% for BC. The assumptions regarding the population structure and selection intensity were the same as described by Nguyen et al. [22]. The economic value for body weight was arbitrarily set to one. The economic value for BC male morphotype was determined iteratively when its genetic gain is zero or −10%. Calculations of selection indices using SelAction [23] showed that without the inclusion of BC the improvement in body weight was 12.5% per generation, and there was an associated increase in BC by 5.3%. By contrast when BC was incorporated into the breeding objective and its genetic gain was restricted to zero (scenario 2), the corresponding gain per generation for body weight was 5.2%. For scenario 3, selection simultaneously improved both traits, that was, increased body weight by 3.5% and decreased BC morphotype by −10%. Our results demonstrated that control of genetic changes in antagonistic characteristics to selection for increased body weight could be effective with some sacrifice in gain for growth performance. However, selection for increase or decrease in a particular male morphotype depends on specific breeding objectives of genetic improvement programs to produce stocks for hatcheries or commercial production. Commercial hatcheries may prefer BC males due to their higher success rate of mating with females than other morphotypes [24], [25]. Results of our present study showed possibilities for the incorporation of male morphotypes in the future genetic improvement program for *Macrobrachium rosenbergii* if that were desired from the industry perspectives.

### Genetic control of female reproductive statuses

In this paper we focused on reporting genetic inheritance of male morphotypes, although female types as categorized by their reproductive statuses were also analysed (results presented in parallel with those of males). The estimates of heritability for female reproductive statuses slightly differed from each other (0.03–0.13 for BF vs. 0.05–0.16 for SF, depending on models). The estimates of heritabilities for female reproductive status were significantly greater than those estimated for morphotypes of males. When body weight of different female classes was treated as different traits, the genetic correlations between homologous trait expressions in female types were very high, close to unity (0.71–0.95). Our results suggest that body weights of female reproductive statuses are essentially the same traits and can be analysed together as shown by Hung et al. [11]. The genetic control of body weights in females is thus likely different from that of males. This is supported by the higher estimates of heritability for body weight of female types than those for males, which is consistent with our earlier report [11]. Our current results together with those reported earlier suggest that body weight of female reproductive status could be analysed together, whereas body weight of males should be treated as different traits to increase accuracy of selection in selective breeding programs for *M. rosenbergii*.

In summary, routine data recording of male morphotypes and female reproductive status should be made to obtain unbiased genetic parameter estimates and increase accuracy of selection and consequently genetic gain. Hung et al. [11] showed that by excluding the effect of morphotype nested within its sex, the estimates of heritability for body weight were overestimated by 58.8%. The inclusion of morphotype effect in statistical models for genetic evaluation of body traits is as described by Hung et al. [11], that is, fitting male morphotypes in male and female reproductive status in female as fixed effects in statistical models for genetic parameter estimation and evaluation. Alternatively body weight of male morphotypes should be treated as genetically different traits as demonstrated in the present study. Separate analyses of male morphotypes are expected to result in higher accuracy of selection and greater genetic gain. They also added to our understanding with regard to possible sources of variation in body traits as well as their genetic variation and association with male morphotypes. Our present study showed that there is potential to change adult male population structure of this species in a preferred direction. Managing the adult male morphotype population structure and agonistic behaviour can be in theory combined with single sex (all male) grow-out cohorts to increase total biomass yield and hence significantly impact farmer returns. There are also possibilities to select for both the ability of each individual to grow, and for reduced competitive behaviour in selection programs for GFP. Selection to improve uniformity of harvest weight is also expected to reduce competition effects if there is genetic variation in residual variance. These areas of research thus deserve considerations for future studies in this unique aquatic species *Macrobrachium rosenbergii*.

## Conclusions

In the present study we demonstrated that male GFP morphotypes exhibit heritable (additive genetic) variation, giving scope for selection to change population structure in this species. Body weight of male morphotypes should also be treated as genetically different traits. The genetic correlations between SM morphotype and growth traits were negative but those between weight (or length) and other morphoptyes (OC, BC, OBC) were positive. Selection for high growth is likely to result in reduced proportion of SM and increased OC (i.e. favourable) but increased proportions of BC and OBC (i.e. unfavourable). Therefore in GFP, male morphotypes should be included in the recording scheme, the breeding objectives and selection indices. A desired gain selection index approach showed that alternative breeding objectives can be developed to simultaneously improve both performance and desired male morphotypes. In freshwater prawn or crustacean species, body length seems to be a good alternative selection criterion to body weight since this trait in GFP male morphotypes may be controlled by a similar set of genes. In contrast to body weight, when body length for male morphotypes were analysed separately, the genetic correlations between the expressions of body length in male morphotypes were close to one (results not presented). Although our present study provided basic quantitative genetic parameters regarding the inheritance of male morphotypes and their body weights, further work is needed to understand biology, physiology and molecular genetics of hierarchical population structure in this species to improve efficiency of practical genetic improvement programs.
